# Excitation of Asymmetric Resonance with Symmetric Split-Ring Resonator

**DOI:** 10.3390/ma15175921

**Published:** 2022-08-26

**Authors:** Ibraheem Al-Naib, Ijlal Shahrukh Ateeq

**Affiliations:** Biomedical Engineering Department, College of Engineering, Imam Abdulrahman Bin Faisal University, Dammam 31441, Saudi Arabia

**Keywords:** split-ring resonator, metamaterials, rectangular waveguide, Fano resonance, sensing

## Abstract

In this paper, a new approach to excite sharp asymmetric resonances using a single completely symmetric split-ring resonator (SRR) inside a rectangular waveguide is proposed. The method is based on an asymmetry in the excitation of a symmetric split-ring resonator by placing it away from the center of the waveguide along its horizontal axis. In turn, a prominent asymmetric resonance was observed in the transmission amplitude of both the simulated results and the measured data. Using a single symmetric SRR with an asymmetric distance of 6 mm from the center of a rectangular waveguide led to the excitation of a sharp resonance with a *Q*-factor of 314 at 6.9 GHz. More importantly, a parametric study simulating different overlayer analytes with various refractive indices revealed a wavelength sensitivity of 579,710 nm/RIU for 150 μm analyte thickness.

## 1. Introduction

Three-dimensional (3D) metamaterials (MMs) [1,2] and two-dimensional (2D) metasurfaces (MSs) [3,4,5] have attracted a lot of attention from scientists and engineers alike due to the unique optical properties that they offer. Conventional MMs consist of a 3D array of unit cells, where a sub-wavelength dimension resonant element is placed in each unit cell. A plethora of configurations have been proposed for various applications across the electromagnetic spectrum [6,7,8,9,10]. The MSs, however, consist of a 2D array of resonant elements, where each element is placed in a unit cell. Conventional MSs typically offer low-quality (*Q*)-factor symmetric dipole mode resonances due to the in-phase excitation of the current distribution in the resonant elements that are normally symmetric with respect to the excitation electric field. Nevertheless, the sharpness of such a mode can be enhanced via the coupling of the nearby resonators [11]. Moreover, it has been shown that the inductive-capacitive (*LC*) mode sharpness can be increased by tuning the periodicity of the MSs to match the first order of the diffractive lattice mode [12]. Furthermore, asymmetric resonance modes have been excited using symmetric resonators by applying strategies using four rotated resonators or applying the analyte to half of the resonators [13,14,15]. Nevertheless, for different applications, such as biosensors, and for various biomolecules like glucose concentration levels in the blood [16,17,18,19,20], as well as for narrow-band filters [21], high *Q*-factor resonances are desirable in order to achieve the required sensitivity and out-of-band rejection. To this end, scientists have proposed different strategies. The first approach is borrowed from Fano-like asymmetric resonance, which occurs when a discrete localized state is coupled to a continuum of states [22,23,24,25,26,27]. It is implemented by breaking the geometrical symmetry of symmetrical resonators with respect to the field excitation [28,29,30]. As one of the applications of such resonators is sensing, another strategy has been suggested to break the symmetry by applying the analyte to half of the resonators instead of the whole structure [14,31]. The second approach is called electromagnetic induced transparency via the coupling of a subradiant component with a radiative component [15,32,33,34,35,36,37,38]. The third approach achieved a quite high *Q*-factor through the conductive coupling of each of the two SRRs in the nearby unit cell [39]. A fourth approach is based on exciting toroidal resonances [23,40,41]. Moreover, it has been shown that various supercells can support quite high *Q*-factors [13,42,43,44]. Various designs have been proposed across the electromagnetic spectrum based on the above approaches, utilizing conventional metal resonators on a dielectric substrate with electric field excitation or complementary structures with magnetic field excitation following the Babinet principle [45,46]. Furthermore, the excitation of symmetric and asymmetric resonances was reported not only using metasurfaces with a large number of resonators, but also with a single resonator inside a rectangular waveguide system [47,48,49]. As a result of the coupling between the resonator inside the waveguide and its mirror images that were enabled by the metallic walls of the waveguide, the collective behavior of the full metasurface was achieved [50,51,52]. Moreover, the system was very compact, and the measurements were more robust compared with a full metasurface.

In this paper, we report the excitation of asymmetric resonance using a completely symmetric SRR with respect to the incident electric field. A single split-ring resonator was strategically placed away from the middle of the C-band waveguide along the horizontal axis, resulting in an imbalance in the excitation of the two arms of the SRR. Hence, the symmetry of the electric field excitation of the SRR was broken, as opposed to the geometry of the SRR itself. A prominent resonance was simulated with full-wave simulations, and was experimentally verified. Below, the electric field and current distribution of the symmetric and asymmetric excitation of the SRR are discussed, along with the wavelength sensitivity. Therefore, the merits of this design are as follows: (i) it uses a single SRR rather than a complete array of SRRs; (ii) it employs a symmetric structure that is not prone to fabrication imperfections; (iii) it utilizes a rectangular waveguide to avoid environmental interference; (iv) it makes it possible to excite sharply asymmetric resonances with large *Q* factors; and finally, (v) it achieves very high wavelength sensitivity.

## 2. Materials and Methods

**Figure 1:**
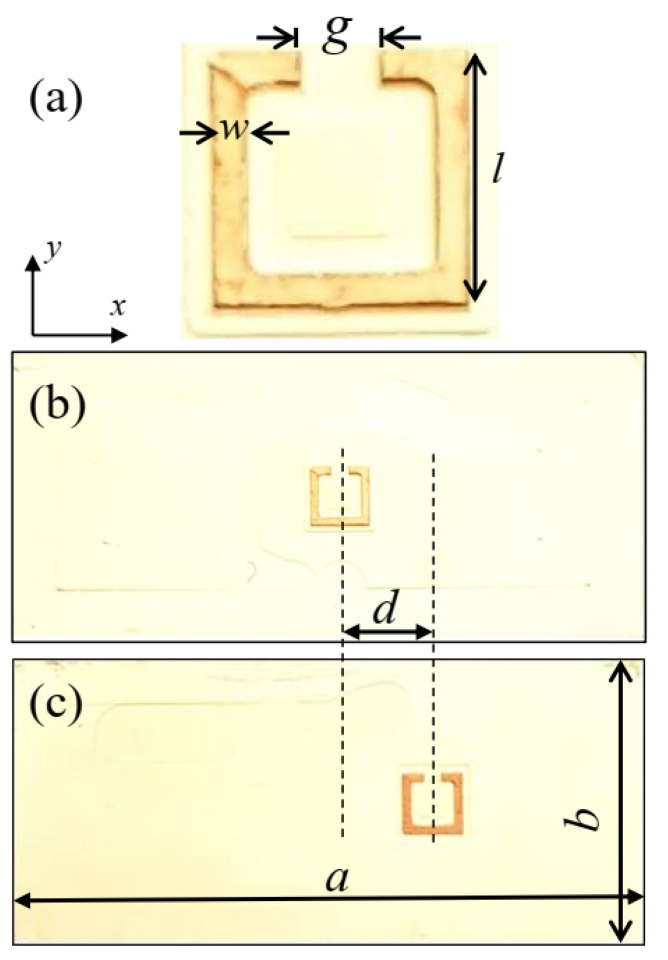
Photos of the fabricated samples. (**a**) A split-ring resonator (SRR) with the important geometrical dimensions, (**b**) Symmetric configuration with the SRR structure in the middle of the waveguide, and (**c**) Asymmetric configuration with the SRR structure shifted by a distance *d*.

A schematic layout of the split-ring resonator with its geometrical dimensions is shown in Figure 1a. In order for the structure to operate in the C-band, i.e., the resonance frequency between 4 GHz and 8 GHz, the dimensions of the resonator were as follows: side length *l* = 3.3 mm, width *w* = 0.6 mm, and gap *g* = 1 mm. A Rogers TMM10 substrate with a dielectric constant of e_r_ = 9.2 was used to fabricate the designed structures with a thickness of 1.27 mm using LPKF prototyping system ProtoMat S63.

The proposed designs were developed such that the x-axis depicted in Figure 1 would be parallel to the longer dimension of the rectangular waveguide. The size of the substrate of each sample was as follows: a width of *a* = 34.85 mm and a height of *b* = 15.8 mm. In this way, the fabricated structures could fit perfectly inside the rectangular waveguide WR137 at the C-band. Figure 1b shows the symmetric structure with the SRR placed exactly in the middle of the substrate, i.e., it was symmetrically excited by the electric field from the waveguide port. Figure 1c depicts the same SRR with the same dimensions and orientation shifted by an asymmetric distance of *d*. Therefore, it was asymmetrically excited by the electric field inside the waveguide that will be discussed later. All the configurations with different asymmetric distances were designed and simulated utilizing the time-domain solver of the 3D electromagnetic CST Microwave Studio wave simulator. The WR137 waveguide with four conducting walls and its real dimensions were considered in the simulation. Furthermore, waveguide ports were employed to excite the designed configurations. Finally, in order to get converged results, an autoregressive filter with adaptive mesh refinement was utilized in the simulator. The experimental part of the study utilized a vector network analyzer, i.e., the FieldFox model N9916A, and a thru-reflect-line calibration kit to calibrate the WR137 rectangular waveguides. After finalizing the calibration, and for each measurement set, a bare dielectric substrate was measured, and its transmission coefficient was used as a reference.

## 3. Results

**Figure 2:**
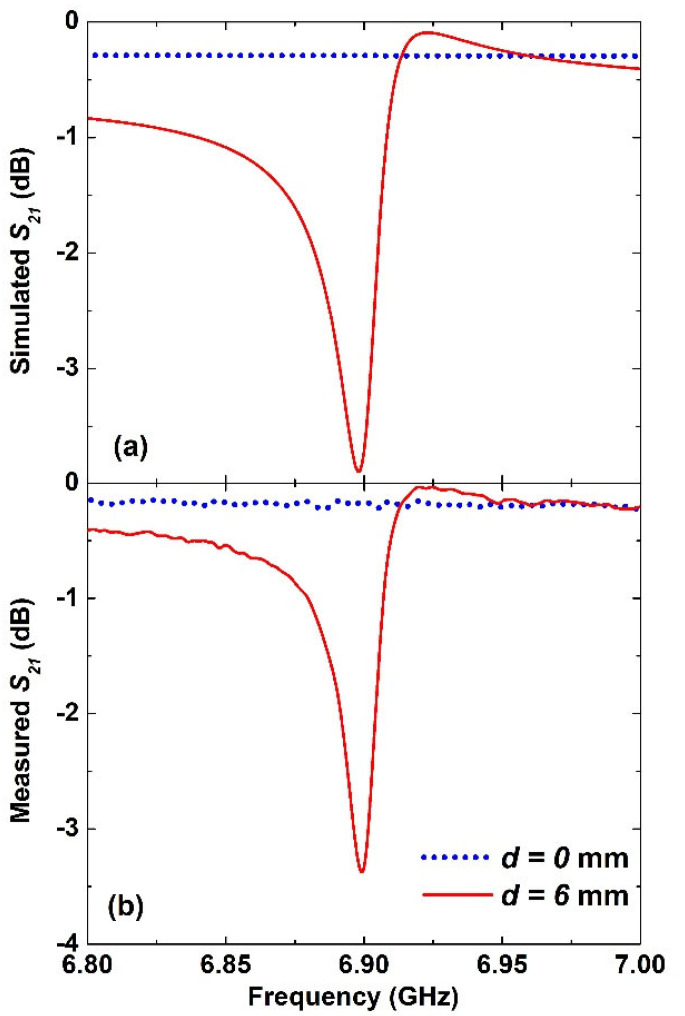
(**a**) Simulated and (**b**) measured normalized transmission amplitude coefficient (*S_21_*) for the symmetric configuration at *d* = 0 (dotted blue lines) and the asymmetric configuration with *d* = 6 mm (solid red lines).

Figure 2a,b show the simulated and measured transmission amplitude, respectively. More specifically, it shows the results for the completely symmetric configuration with respect to the excitation when the asymmetric distance *d* = 0 mm, as depicted in Figure 1b with dotted blue lines. Meanwhile, the results of the asymmetric configuration when the asymmetric distance *d* = 6 mm that is depicted in Figure 1c are shown in Figure 2 as solid red lines.

When the split-ring resonator was exactly in the middle of the waveguide, the transmission spectral response shown as dotted blue lines was rather flat, with a very small insertion loss of about 0.2 dB for the frequency range between 6.8 GHz and 7 GHz. There was no sign of any resonance excitation as the SRR was symmetrically excited and the only possible resonance was the dipole mode with a frequency that was outside the above frequency range. Remarkably, there was a prominent resonance at 6.9 GHz when the same split-ring resonator with the same dimensions and orientation was moved to the right along the x-axis by 6 mm. Its simulated and experimentally measured transmission amplitude coefficients are shown with solid red lines in Figure 2a and Figure 2b, respectively. This resonance was excited as a direct effect of the asymmetric excitation of the split-ring resonator, i.e., not by breaking its symmetry at all. Interestingly, there was very good agreement between the simulated and measured data, with the transmission amplitudes at the resonance reaching −4 dB in the simulation and −3.5 dB in the measurements. Similarly, the full-width-half maximum bandwidth was about 28 GHz in the simulated results and 22 MHz from the measurements data.

Hence, the achieved *Q*-factor of the measured data, defined as the ratio of the resonance frequency to the bandwidth, was 314. This value could be enhanced using a very low loss-tangent of the substrate dielectric material and better conductivity of the metallic layer. Nevertheless, the achieved *Q*-factor was quite high, given the fact that this configuration was a metasurface with a thickness of 35 μm only. A much larger *Q*-factor could be reached if multiple layers of the same structure were successively arranged with a suitable separation in the direction of propagation. Moreover, the exciting resonance revealed an asymmetric response, and hence, it indicated that the current distribution at this resonance was out-of-phase. There was, of course, a dipole resonance of the SRR structure that was excited beyond the frequency range shown here.

**Figure 3:**
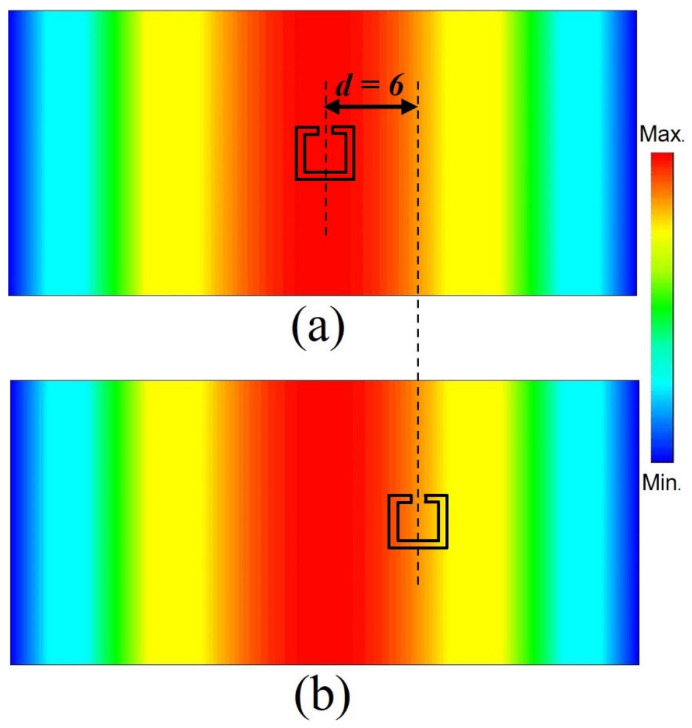
Distribution of the waveguide port electric field mapped with the (**a**) symmetric and (**b**) asymmetric configurations of the designed structures.

In order to understand the symmetrical and asymmetrical excitation of the developed proposed configuration, Figure 3 shows the rectangular waveguide port electric field distribution with the designed SRR structures mapped on the top of the field distribution. In Figure 3a, the split-ring resonator is mapped exactly at the middle of the electrical field, i.e., at *d* = 0; it can be seen from the figure that it was symmetrically excited with respect to the distribution of the electrical field. In this case, in-phase only current distribution was excited in the right and left parts of the SRR, and hence, only the dipole mode could be excited. Similarly, in Figure 3b, the same split-ring resonator was moved to the right by 6 mm from the center of the waveguide. Therefore, the electric field asymmetrically excited the SRR.

**Figure 4:**
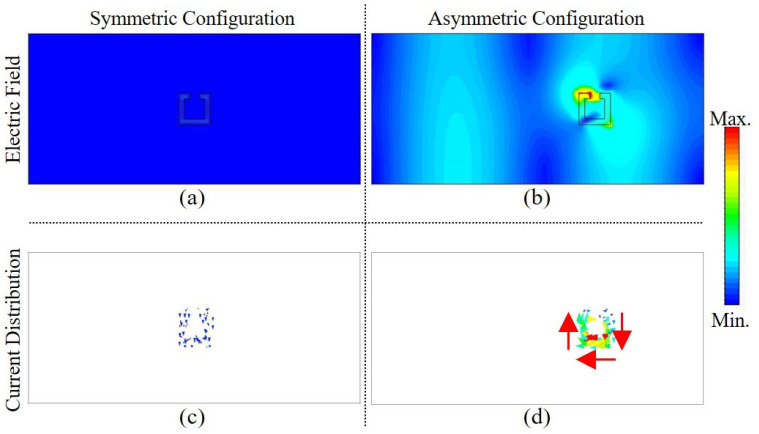
(**a**,**b**) Spatial electric field distribution at the surface of the designed SRR structures at 6.9 GHz for the symmetric and asymmetric configurations, respectively; (**c**,**d**) current distribution at 6.9 GHz for the same configurations. The red arrows were added to visualize the direction of the current.

In order to explain the physical mechanism of the proposed structure, further simulations were carried out to calculate the electric field and the corresponding current distribution for both the symmetric and asymmetric configurations at the resonance frequency. Figure 4a,c depict the results of the symmetric configuration and Figure 4b,d display the results of asymmetric configuration. For the former, the electric field shown in Figure 4a was at a minimal value and the corresponding current distribution shown in Figure 4c was very weak. Conversely, for the asymmetric case when the asymmetric distance *d* = 6 mm, the electric field was very well confined with high values near the gap of the SRR, as shown in Figure 4c. Most importantly, the current distribution of the case shown in Figure 4d featured an in-phase behavior and its strength was much larger than its counterpart with a symmetric configuration. It is worth mentioning that with the split in the right or left arm of the resonator instead of the top side, and without even shifting the resonator, the structure will be asymmetric with respect to the incident electric field. Hence, the famous inductive-capacitive (*LC*) resonance mode was excited, along with the dipole resonance mode.

**Figure 5:**
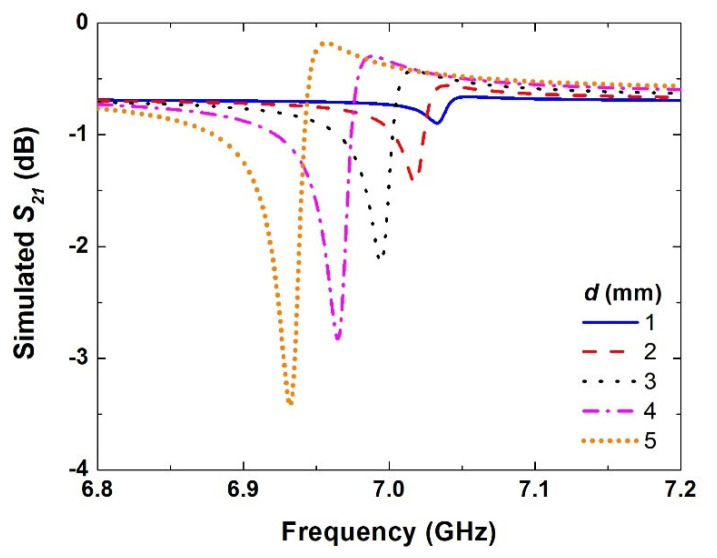
Simulated transmission amplitude coefficient (*S*_21_) of designed SRR structures at different asymmetric distances (*d*) from the center.

Next, a series of simulations was carried out by moving the SRR from the center of the rectangular waveguide to the right with different asymmetric distances (*d*), i.e., from 1 mm to 5 mm, with a step of 1 mm, as demonstrated in Figure 5.

It can be seen from these results that as soon as the SRR had been moved by 1 mm, the asymmetric resonance was excited, albeit with a very small amplitude, because the out-of-phase current was quite small. As the asymmetric distance was further increased, there was a clear increase in the amplitude of excited resonance, as well as the corresponding bandwidth. This could be attributed to the asymmetric excitation of the SRR leading to a difference in the electrical length of two arms of the SRR, which led to a small shift in the resonance frequency. As presented in Figure 2, an asymmetric distance of 6 mm was chosen as it offers quite good sharpness and a suitable resonance amplitude that could easily be measured. Increasing the asymmetric distance beyond that showed a further increase in the bandwidth but did not result in much difference in the resonance amplitude. Hence, an asymmetric distance of 6 mm was chosen in the following sensing evaluation. This kind of study enables the designers of such devices to choose the right asymmetric distance based on the desired amplitude and bandwidth according to the application requirements and the available dynamic range.

**Figure 6:**
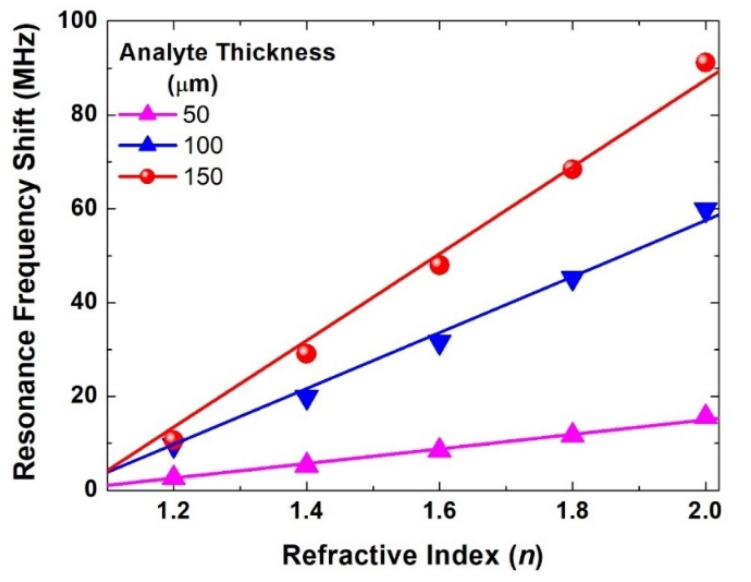
Resonance frequency shift in MHz versus the refractive index (*n*) of the overlayer analyte with different thicknesses between 50 and 150 μm.

One application for such a design could be sensing. For this purpose, it is quite important to evaluate the sensitivity of the design with different analyte thicknesses and refractive indices. Hence, parametric simulations were performed for the asymmetrical configuration with an asymmetric distance of 6 mm with different thicknesses of analyte overlayer, i.e., 50 μm, 100 μm, and 150 μm, for a sweep of its refractive index between 1.2 and 2.0 with a step of 0.2. The results are shown in Figure 6 with a linear fit of the resonance frequency shift of each set of simulations at a given thickness. The slope of these line curves indicates the sensitivity of the design, which is given as a ratio of the resonance frequency shift in Hz by the refractive index unit (RIU). For the simulated thicknesses, the slope of these linear fits was 15.5, 59.7, and 92.6 MHz/RIU for thicknesses of 50, 100, and 150 μm, respectively. However, the exact shift will vary if the SRR structure is designed at a different resonance frequency. Hence, a better metric that can be calculated is wavelength sensitivity, that is given by [53,54]:

(1) $$S=\left|\frac{d\lambda }{dn}\right|=\frac{\Delta f}{\Delta n}\times \frac{c_{o}}{f_{r}^{2}}$$

where Δf is the asymmetric resonance frequency shift, Δn is the refractive index difference, $c_{o}$ is the speed of light, and $f_{r}$ is the resonance frequency. Following this methodology, the wavelength sensitivity was found to be 98,551 nm/RIU, 376,938 nm/RIU, and 579,710 nm/RIU for the three analyte thicknesses. The increase in the wavelength sensitivity by increasing the analyte thickness from 100 μm to 150 μm was about 52% compared to 282% when the analyte thickness increased from 50 μm to 100 μm. Simulations with a larger value of the analyte thickness showed a similar trend, i.e., the resonance shift increased, but there was a clear sign of saturation in its absolute value. Such an effect was expected when a refractive index value greater than 2.0 was used, i.e., as the electric field faded away from the surface of the SRR.

Including the thickness of the analyte in the above equation may provide additional information about the sensitivity. It is worth mentioning that for a fair comparison with other configurations, many parameters have to be considered. This topic was thoroughly discussed in a recent paper [55]. Therefore, in order to provide a fair comparison, a sensitivity analysis was carried out for the asymmetric double split ring resonator (aDSR) that provides excitation of Fano-like resonance, which has been the subject of many papers [28,29,47,49]. The dimensions of the aDSR were optimized such that the resonance frequency was almost the same as that of the current design. The same overlayer thickness, i.e., 150 μm, and the same refractive index, i.e., 2.0, were utilized. The resulting sensitivity was found to be 485,398 nm/RIU. Hence, the wavelength sensitivity of the proposed structure in this paper was 19.4% larger than that provided by the aDSR design. Other aspects that could be considered are the amount of sample or the amount of water in a given sample. It is always desirable to reduce these amounts in order to reduce the associated losses that can lead to the broadening, or even the disappearance, of a resonance. As an example, the authors of [49] successfully used 1 μL glucose solution to differentiate between different glucose level concentrations.

## 4. Conclusions

In conclusion, a unique single SRR-waveguide configuration that mimics a 2D metasurface that operates in C-band has been proposed in this paper. It is based on a new approach to exciting sharp asymmetric resonance based on asymmetry via the excitation of completely symmetric SRR by placing it away from the center of a rectangular waveguide. The results of the fabricated design, measured using a Rogers TMM10 substrate, matched the simulated transmission amplitude quite well. Moving the SRR from the middle of the rectangular waveguide by 6 mm to the right led to the excitation of a very clear asymmetric resonance at 6.9 GHz with a bandwidth of 22 MHz and a *Q*-factor of about 314. Moreover, the electric field and the current distributions were simulated and discussed to examine the physical mechanism behind the excitation of this resonance. Furthermore, the wavelength sensitivity for different analyte thicknesses was evaluated. Values as high as 579,710 nm/RIU for 150 μm analyte thickness were numerically demonstrated. In the future, such a compact and well-controlled SRR-waveguide configuration may pave the way for applications such as narrowband filtering and biosensors.

## Data Availability

Not applicable.
